# Birt-Hogg-Dubé Syndrome: Two Patients With Different Initial Presentations

**DOI:** 10.7759/cureus.30578

**Published:** 2022-10-22

**Authors:** Bibek Bakhati, Genesis Perez Del Nogal, Ivania Salinas, Kelash Bajaj

**Affiliations:** 1 Internal Medicine, Texas Tech University Health Sciences Center, Odessa, USA; 2 Oncology, Texas Oncology, Odessa, USA

**Keywords:** tumor suppressor gene, rare genetic disorder, tumor imaging, renal cell carcinoma (rcc), birt-hogg-dubé syndrome

## Abstract

Birt-Hogg-Dubé syndrome (BHD) is a rare genetic disorder caused by germline mutations in the tumor suppressor folliculin gene (FLCN). This condition is characterized by benign skin hamartomas, pulmonary cysts, spontaneous pneumothorax, and an increased risk for developing kidney tumors which range from benign oncocytomas to malignant renal cell carcinomas including chromophobe, clear cell, or papillary subtypes. We describe two cases of BHD with different initial presentations. Patients underwent genetic testing and an FLCN mutation was identified, confirming the diagnosis. Through this case series, we aim to highlight the importance of recognizing key manifestations of BHD whether alone or in combination, followed by genetic testing and counseling and the need for regular follow-ups with surveillance imaging tests to detect renal cancer early on.

## Introduction

Birt-Hogg-Dubé syndrome (BHD) is an autosomal dominant condition caused by germline mutations in the tumor suppressor folliculin gene (FLCN). This syndrome is characterized by benign skin hamartomas, most commonly located on the head and neck, pulmonary cysts, spontaneous pneumothorax, and an increased risk of renal cancer [1]. The incidence of BHD syndrome is unknown. Approximately 400 families have been identified worldwide [2].

## Case presentation

Case 1

A male patient in his 60s with a past medical history significant for cystic lung disease, repetitive pneumothorax during his adolescence, and prostate cancer was treated with brachytherapy 14 years ago. He presented to the emergency department (ED) complaining of intermittent hematuria and left flank pain for two weeks. He denied any recent trauma, fever, chills, dysuria, tenesmus, frequency, diarrhea, dizziness, fatigue, weakness, or weight loss. Regarding his family history, his mother was diagnosed with breast cancer at age 66, thyroid cancer at age 68, and renal cancer at age 76. His father was diagnosed with lung cancer at 68, and his son had a history of pneumothorax. Social history was relevant for tobacco use with a 10-pack-year history.

On examination, vital signs were stable. The abdomen exam was positive for left lower quadrant tenderness. General examination including skin was unremarkable.

**Figure 1:**
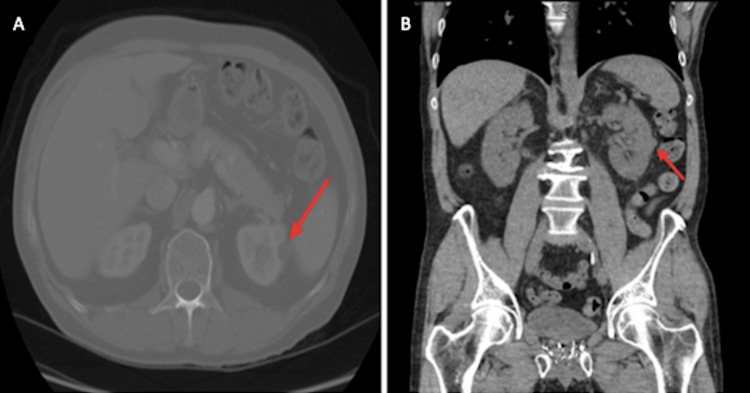
Contrast-enhanced CT abdomen and pelvis CT abdomen in cross-sectional (Panel A) and longitudinal (Panel B) view showed poorly defined solid left renal mass suspicious for renal cell carcinoma (pointed by red arrow).

Laboratories on admission evidenced normal hemoglobin of 14.6 g/dL, white blood cell count 6.3 x 10^3^/uL, platelets 287 x 10^3^/uL, creatinine 1 mg/dL, blood urea nitrogen (BUN) 18 mg/dL, with normal electrolytes. Macroscopic hematuria was noted, and 197 red blood cells per high-power field were present on urinalysis. A contrast-enhanced CT abdomen was ordered and showed a poorly defined solid left renal mass suspicious for renal cell carcinoma, accompanied by a normal-looking right kidney and bladder (Figure 1).

**Figure 2:**
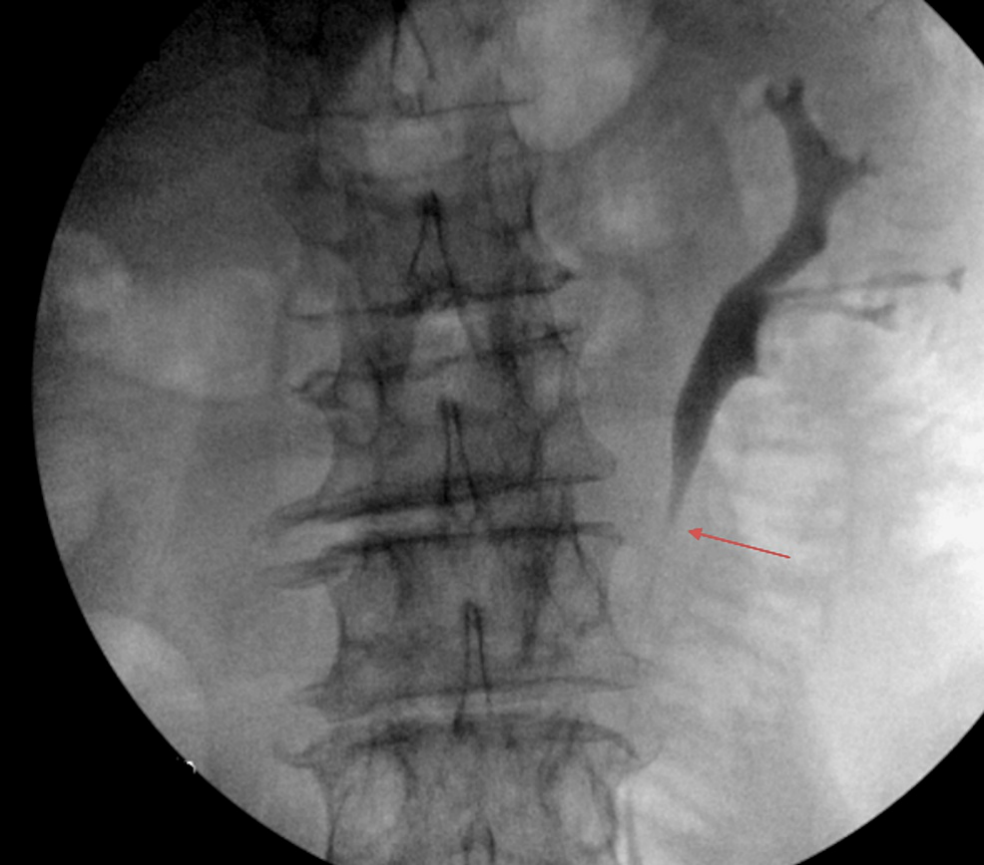
Left retrograde pyelogram Left retrograde pyelogram evidenced filling defect (pointed by red arrow).

Urology was consulted and a cystoscopy, with left retrograde pyelogram and left ureteroscopy with biopsy were performed (Figure 2). Biopsy proved to be positive for renal cell carcinoma with tubular papillary structures.

**Figure 3:**
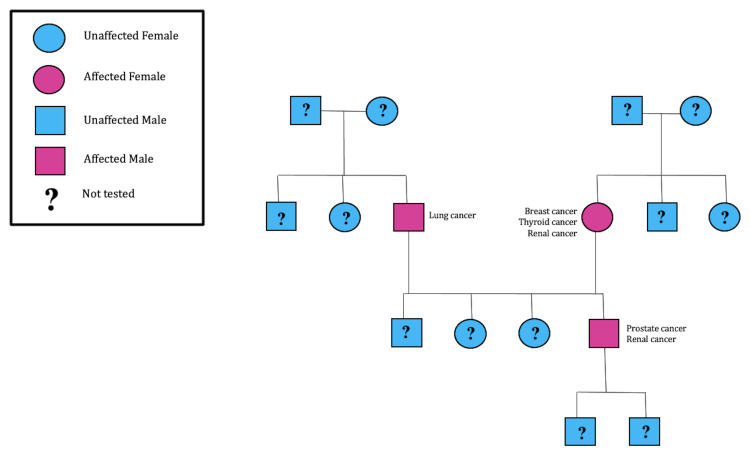
Pedigree chart of the patient’s family (Case 1)

Due to the newly diagnosed papillary renal cell carcinoma, and significant family history of cancer (Figure 3), the patient was referred for genetic counseling, and laboratories detected heterozygous, germline mutation on the Folliculin (FLCN) gene, consistent with the diagnosis of Birt-Hogg-Dube syndrome. Eventually, the patient had a left radical nephrectomy.

Nonetheless, a few months after surgery the patient presented with new-onset back pain. To rule out metastatic disease, multiple imaging studies were ordered. Magnetic resonance imaging (MRI) of the brain showed small sequelae of remote infarcts in the basal ganglia, greater on the left side, however, there was no evidence of metastatic disease. MRI of the spine demonstrated moderate cervical degenerative changes along with findings consistent with osseous metastasis involving thoracic vertebral bodies. Fluoroscopic-guided biopsy showed poorly differentiated carcinoma suggesting metastasis from the renal tumor. Consequently, the patient started radiotherapy but decided to discontinue treatment after new-onset dysphagia, which he considered was related to the treatment.

Case 2

An adult male in his 60s was referred to oncology for cancer risk assessment. He initially presented to his dermatologist for multiple facial skin lesions which had been removed. A biopsy of a right postauricular skin lesion showed fibrofolliculoma with a junctional nevus. Due to the association of such lesions with BHD, he was referred for further assessment. At the time of the presentation, he denied any major concerns. His medical history was significant for hypertension, type 2 diabetes, morbid obesity, and chronic back pain for which he underwent lumbar discectomy twice with subsequent placement of a spinal cord stimulator. He denied any perioperative complications, history of spontaneous pneumothoraces, pulmonary cysts, breathing difficulties, flank pain, hematuria, or constitutional symptoms. Except for social drinking, he denied smoking tobacco products or using illicit drugs. Family history was positive for smoking-associated lung cancers. Vital signs upon assessment were stable and physical examination was unremarkable.

**Figure 4:**
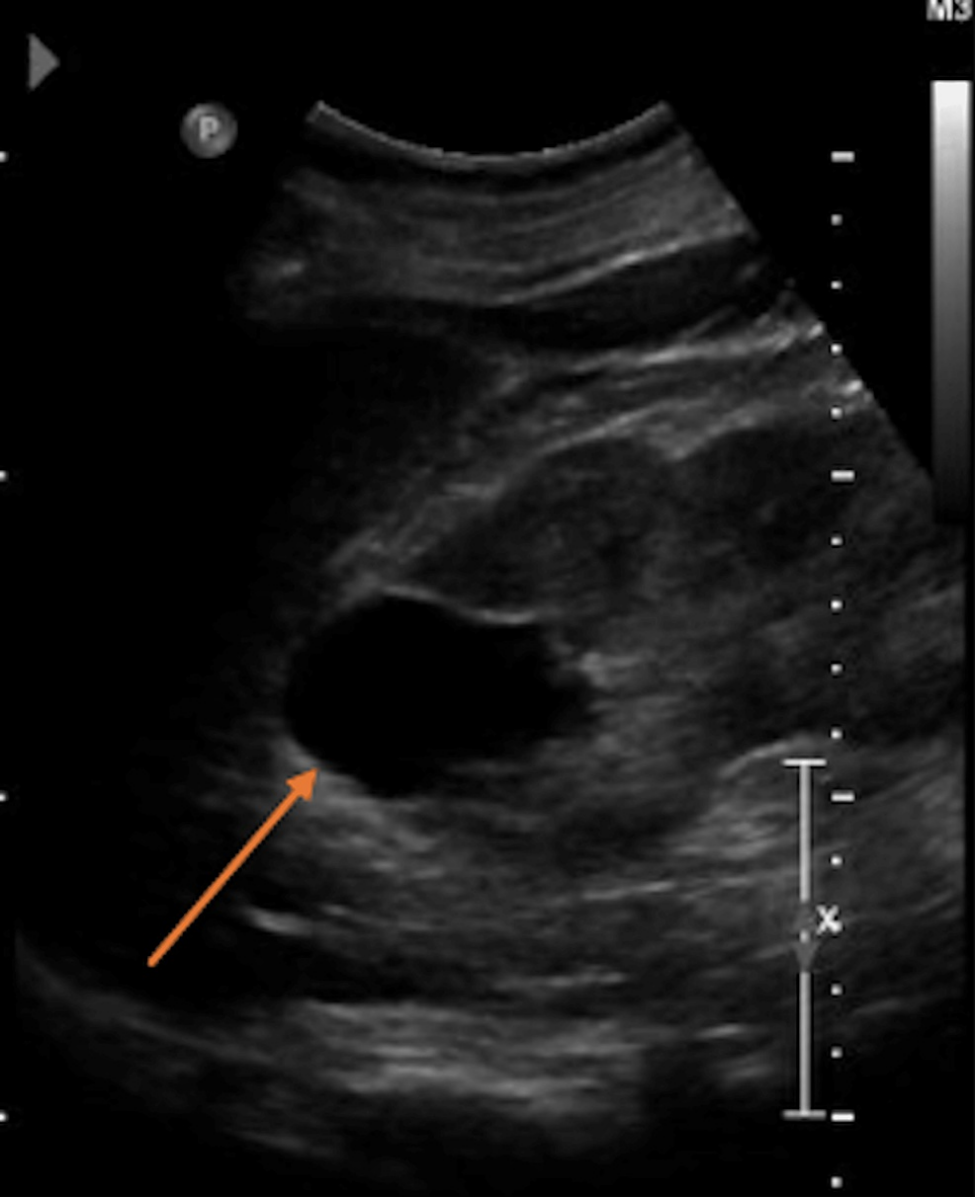
Renal ultrasound Renal ultrasound showed a 4.8 x 3.0 x 3.0 cm thin-walled cyst in the upper pole of the left kidney (orange arrow).

Assessment for FLCN gene mutation was done, and a pathogenic variant was identified [Exon 12, c.1429C>T (p.Arg477*), heterozygous]. Consult for genetic counseling was requested and he underwent renal ultrasound which revealed a 4.8 x 3.0 x 3.0 cm thin-walled cyst in the upper pole of the left kidney (Figure 4).

**Figure 5:**
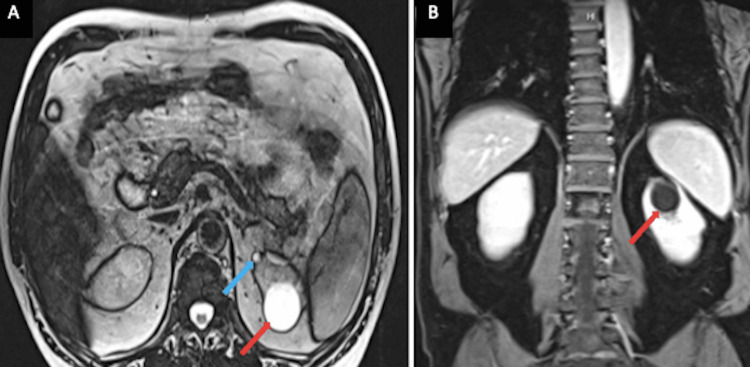
MRI of the abdomen MRI of the abdomen in cross-sectional (Panel A) and longitudinal (Panel B) view showed a benign-appearing simple left renal cyst (red arrows) along with another tiny simple cyst measuring 9 mm more anteriorly in the upper pole of the left kidney (blue arrow in panel A).

Subsequent MRI of the abdomen with and without contrast approximately seven months later demonstrated previously visualized benign-appearing simple left renal cyst along with another tiny simple cyst measuring 9 mm in maximum diameter more anteriorly in the upper pole of the left kidney without suspicious renal lesions (Figure 5).

## Discussion

Birt-Hogg-Dubé (BHD) syndrome was first described by the respective scientists in 1977 and is characterized by noncancerous cutaneous lesions, multiple pulmonary cysts, spontaneous pneumothoraces, and varied renal tumors [1,3]. Germline mutations in the tumor suppressor gene, Folliculin (FLCN), are responsible for the condition, and the locus is located on chromosome 17p11.2 [4,5]. It encodes folliculin tumor suppressor protein which interacts with folliculin-interacting protein 1 (FNIP1) and folliculin-interacting protein 2 (FNIP2). They interact with 5′ AMP-activated protein kinase (AMPK) which negatively regulates the mammalian target of rapamycin (mTOR) and modulates the AKT-TOR signaling pathway [4,6,7].

Fibrofolliculomas, trichodiscomas, and acrochordons are the classic cutaneous manifestations of BHD [3]. Fibrofolliculomas are the most common and develop after the age of 20 years, as was noted in the second patient of the case series [8]. Fibrofolliculomas and trichodiscomas are benign hair follicle tumors called follicle hamartomas [9]. They are clinically identical and present as small, round, dome-shaped, whitish lesions mainly on the facial region, neck, and upper part of the trunk [3,8,9]. On rare occasions, angiofibromas may be associated with BHD but one must maintain a high index of suspicion for Tuberous Sclerosis (TM) in case of multiple facial angiofibromas [3,8].

Pulmonary cysts and spontaneous pneumothoraces are also characteristic of BHD [4]. Multiple pulmonary cysts can be detected on a computed tomography scan (CT) of the thorax in more than 80% of the affected population [1]. These cysts tend to be located largely in the bases and medial portions of the lungs as opposed to the typical apical distribution in patients with emphysema and primary spontaneous pneumothorax [10]. Per Zbar et al., the odds ratio (OR) for developing spontaneous pneumothorax was 50.3 in the BHD population [11]. Our first patient had a history of cystic lung disease and repetitive spontaneous pneumothoraces during his adolescence.

The most concerning finding in BHD is renal tumors and they tend to be multiple, bilateral, and are diagnosed on average at 50 years of age [1]. In contrast to other hereditary renal cell carcinomas (RCC) syndromes, renal cancers in BHD are less likely to be aggressive and have diverse histopathologic subtypes [1,8]. Multivariate analysis showed that the odds of developing kidney tumors were 6.9 times higher in BHD patients [10]. A pathological review of 130 renal tumors from 30 BHD patients by Pavlovich et al. revealed that hybrid oncocytic tumor constituted 50%, chromophobe RCC 34%, clear cell RCC 9%, oncocytoma 5%, and papillary RCC 2% (Table 1) [12]. Our patient 1 was found to have papillary RCC.

**Table 1:** The histology of renal tumors in BHD BHD: Birt-Hogg-Dubé syndrome, RCC: Renal cell carcinoma.

The histology of renal tumors in BHD	
Hybrid oncocytic tumor	50%
Chromophobe RCC	34%
Clear-cell RCC	9%
Oncocytoma	5%
Papillary RCC	2%

The diagnosis of BHD should be considered based on personal and/or family history of above mentioned cutaneous, pulmonary, and renal findings, alone or in combination [3,4]. Confirmatory diagnosis can be made with the help of a genetic test demonstrating FLCN germline mutation [4]. Identifying pathogenic FLCN germline variants is important for definitive diagnosis especially in those with only pulmonary and/or renal manifestations, as it was in the case of patient 1, where he had no cutaneous presentation [3]. It is also worth noting that in the event of negative genetic testing, multiplex ligation-dependent probe amplification (MLPA) and next-generation sequencing (NGS) may need to be considered [13].

Due to the noncancerous nature of the skin lesions, they usually don’t require treatment unless for cosmetic reasons, and pneumothorax is treated the same way as in the general population [8]. However, due to increased susceptibility to developing spontaneous pneumothoraxes, BHD patients should be counseled to avoid smoking, radiation, and activities related to high atmospheric pressure such as scuba diving [8,14].

Surveillance for renal tumors is recommended with abdominal magnetic resonance imaging (MRI) or computerized tomography (CT) from age 21 since the ultrasonogram fails to detect small masses. Also, they should be followed every three years until the imaging tests identify renal lesions [14]. Surgery is the keystone to the treatment of renal cancers in these patients, and nephron-sparing surgery is generally recommended once they are larger than 3 cm [8,14]. Genetic counseling and surveillance follow-up imaging tests should be offered to all at-risk individuals once a proband has been identified [8].

In our cases, both patients were referred for genetic counseling and an FLCN mutation was identified. Our first patient had left-sided papillary RCC and underwent a radical nephrectomy. Unfortunately, he was found to have metastatic lesions to the vertebrae during subsequent follow-ups. Patient 2 only had fibrofolliculoma without concerning renal lesions in surveillance imaging tests afterward.

## Conclusions

Despite the classic triad, BHD patients may not present with all the manifestations. Genetic counseling and regular follow-ups with imaging tests should be offered to BHD patients due to increased risk of renal cancer. Surgery is the cornerstone for the treatment of renal masses, and it should be offered once they are larger than 3 cm. Nephron-sparing surgery is generally preferred.
